# Changes in Arterial Stiffness Monitored Using the Cardio-Ankle Vascular Index in Patients with Rheumatic Disease Receiving Initial Glucocorticoid Therapy: A Clinical Pilot Study

**DOI:** 10.3390/jcm12216923

**Published:** 2023-11-03

**Authors:** Kaichi Kaneko, Daiki Sakai, Shuji Sato, Toshio Kinoshita, Kazuhiro Shimizu

**Affiliations:** 1 Division of Rheumatology, Department of Internal Medicine, Toho University Sakura Medical Center, 564-1 Shimoshizu, Sakura 285-8741, Chiba, Japan; ihciakokenak@med.toho-u.ac.jp (K.K.); daiki.sakai@med.toho-u.ac.jp (D.S.); 2 Division of Cardiovascular Medicine, Department of Internal Medicine, Toho University Sakura Medical Center, 564-1 Shimoshizu, Sakura 285-8741, Chiba, Japan; shuuji.satou@med.toho-u.ac.jp (S.S.); kino-00039@med.toho-u.ac.jp (T.K.)

**Keywords:** arterial stiffness, CAVI, glucocorticoid, lymphocyte/monocyte ratio, rheumatic diseases

## Abstract

Systemic inflammatory rheumatic diseases predispose to premature birth, accelerated atherosclerosis, and increased cardiovascular disease (CVD). While glucocorticoids (GCs) are used in various rheumatic diseases, and the associations between GC excess and increased prevalence of CVD complications are well established, the mechanisms underlying GCs’ role in atheroma development are unclear. We conducted an observational study to address GC therapy’s effect on arterial stiffness using the cardio-ankle vascular index (CAVI) in patients with rheumatic diseases. Twenty-eight patients with rheumatic disease received initial GC therapy with prednisolone at doses ranging from 20 to 60 mg/d. CAVI was examined at baseline and 3 and 6 months after GC therapy. Changes in CAVI and inflammatory parameters were evaluated. GC therapy increased the mean CAVI after 3 months but decreased it to pretreatment levels after 6 months. The mean CAVI substantially decreased with GC treatment in patients <65 years but increased in patients ≥65 years. Alterations in CAVI during the 6-month GC treatment negatively correlated with the lymphocyte-to-monocyte ratio (LMR) at baseline. Conversely, no correlation was observed between alterations in CAVI values and conventional inflammatory markers (C-reactive protein and erythrocyte sedimentation rate). Multivariate analysis of factors related to changes in CAVI highlighted young age, high prednisolone dosage, and LMR at baseline. GC temporarily exacerbates but eventually improves arterial stiffness in rheumatic diseases. Particularly in young patients, GC may improve arterial stiffness by reducing inflammation. Therefore, the LMR before GC therapy in rheumatic diseases may be a potential predictor of arterial stiffness.

## 1. Introduction

Systemic inflammatory rheumatic diseases such as rheumatoid arthritis (RA) and systemic lupus erythematosus (SLE) result in increased cardiovascular morbidity and mortality owing to accelerated atherosclerosis and premature coronary artery disease (CAD) [1,2]. Traditional cardiovascular risk factors, such as hyperlipidemia, hypertension, diabetes mellitus, and aging, do not always explain the increased risk of cardiovascular disease (CVD) associated with systemic rheumatic diseases. The increased risk of CVD in systemic rheumatic diseases is the result of a complex interaction involving disease-associated risk factors, such as inflammation and immune abnormalities, and anti-inflammatory agents, such as glucocorticoids (GCs) [3]. While GCs are the gold standard for reducing immune activation and inflammation in various inflammatory and systemic rheumatic diseases, various side effects of long-term treatment, such as atherosclerosis development, have become a major challenge [3,4,5]. A correlation has been established between chronic GC excess and increased CVD risk, including the emergence of key risk factors, including diabetes, hypertension, and dyslipidemia [6,7]. Atherosclerosis is a chronic inflammatory disease of the arterial wall triggered by traditional and non-traditional risk factors and mediated by inflammatory and immune responses [8]. GC treatment has long been a mainstay for reducing inflammation associated with rheumatic diseases, and these anti-inflammatory interventions may prevent CVD [9].

Recently, the lymphocyte-to-monocyte ratio (LMR) was identified as a novel systemic inflammatory marker [10] and has been examined as a prognostic predictor in various cancers [11]. The LMR, which is widely accessible in clinical practice, has been proven to be an important inflammatory marker and a potential predictor of CVD risk [12]. However, to our knowledge, a relationship between LMR and CVD risk in systemic rheumatic diseases has not been established.

The cardio-ankle vascular index (CAVI) has been established as a marker of arterial stiffening and is associated with atherosclerosis-related diseases, including diabetes, hypertension, and aging [13]. Data regarding alterations in arterial stiffness during GC therapy for rheumatic diseases are lacking. We hypothesized that the degree of arterial stiffness in rheumatic diseases would be decreased by GC treatment and that CAVI could be used to assess alterations in arterial stiffness.

In this study, we investigated the association between changes in CAVI and inflammation over a 6-month follow-up in patients who received GC therapy for rheumatic diseases. The effects of GC therapy on the progression of arterial stiffness were also investigated.

## 2. Materials and Methods

### 2.1. Patients

**Figure 1:**
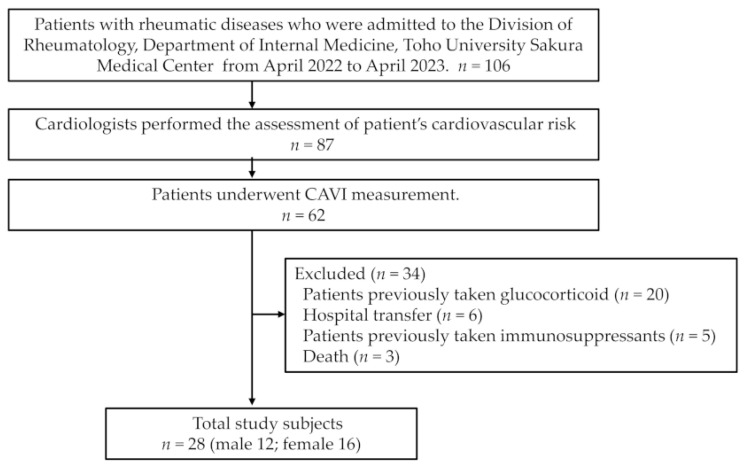
Flow chart of patient selection. CAVI, cardio-ankle vascular index.

This retrospective observational study was conducted using the opt-out method, and approved by the Ethics Committee of the Toho University Sakura Medical Center (approval number: S23014). From the patients with rheumatic diseases who were newly received GC from April 2022 to April 2023 at Toho University Sakura Medical Center, we selected 28 patients who underwent CAVI measurements to monitor cardiovascular complications (Figure 1). The underlying diseases include: vasculitis syndrome (*n* = 5), polymyalgia rheumatica (*n* = 5), SLE (*n* = 3), ImmunoglobulinG4-related disease (*n* = 3), dermatomyositis/polymyositis (*n* = 3), adult-onset Still’s disease (*n* = 3), remitting seronegative symmetrical synovitis with pitting edema syndrome (*n* = 2), systemic sclerosis (*n* = 2), RA (*n* = 1) and relapsing polychondritis (*n* = 1). We included patients with high disease activity who were newly starting treatment with prednisolone at doses of 20–60 mg daily (mean daily dose: 45.2 ± 1.9 mg (SD)). Patients were excluded if they had previously received GCs or other immunosuppressive drugs. Fasting morning blood samples were collected before the patients started treatment and after 3 and 6 months of GC therapy.

### 2.2. Clinical and Laboratory Measurements

Clinical information and laboratory data were obtained from a structured interview, self-reported questionnaires, physical examinations, and blood tests. Body mass index (BMI) was calculated based on measured height and weight. The baseline blood pressure was calculated by taking the average of two readings. The morning blood samples were collected to measure the baseline levels of serum C-reactive protein (CRP) using latex nephelometry (LSI Medience Corp., Tokyo, Japan), total cholesterol, high-density lipoprotein cholesterol (HDL-C) and low-density lipoprotein cholesterol (LDL-C) using the homogeneous method (Sekisui Medical Co., Ltd., Tokyo, Japan), and triglycerides using enzymatic assay (Sekisui Medical Co., Ltd., Tokyo, Japan). Complete blood counts, including lymphocyte, neutrophil, and monocyte counts, were performed for each participant using an automatic blood counting system (Sysmex, Tokyo, Japan). The LMR is the ratio calculated by dividing the absolute lymphocyte counts by the absolute monocyte counts from the blood test.

We assessed the presence or absence of hypertension (defined as hypertension if they had systolic blood pressure (SBP) ≥140 mmHg and/or diastolic blood pressure (DBP) ≥90 mmHg [14,15] or were taking antihypertensive agents), the presence or absence of diabetes mellitus (defined by a HbA1c ≥6.5% [16] or the requirement of antidiabetic agents) and dyslipidemia (defined as low-density lipoprotein cholesterol concentration ≥ 140 mg/dL, high-density lipoprotein cholesterol concentration < 40 mg/dL, triglyceride concentration ≥ 150 mg/dL [17], or requiring antihyperlipidemic treatment) as traditional risk factors for atherosclerosis.

### 2.3. Measurement of CAVI 

CAVI measurements were taken using a VaseraVS-3000 vascular screening system (Fukuda Denshi, Tokyo, Japan) before starting GC therapy and at 3 and 6 months post-treatment, as described by Shirai et al. [13]. The system measured the electrocardiogram, phonocardiograph, and pressures and waveforms of the brachial and ankle arteries and automatically calculated the CAVI using built-in software. CAVI is measured with high measurement reproducibility. Shirai et al. reported the coefficient of variation to be 3.8% [13]. A trained medical laboratory scientist measured the CAVI. CAVI was determined using the following equation: CAVI value = a {(2p/ΔP) × In (Ps/Pd) PWV2} + b, in which Ps and Pd are SBP and DBP in mmHg, respectively; PWV is pulse-wave velocity from the origin of the aorta to the tibial artery-femoral artery junction; ΔP is the difference between SBP and DBP; p is blood density; and a and b are constants. Higher values of left and right CAVI were adopted for analysis.

### 2.4. Statistical Analysis

Statistical analyses were performed using Prism ver. 8.0 (GraphPad Software, San Diego, CA, USA) and EZR on R Commander statistical software ver. 1.61 (Saitama Medical Center, Jichi Medical University, Saitama, Japan). Numerical data were expressed as mean ± SD and the median with the interquartile range. Changes during GC treatment were assessed using Friedman’s test, followed by Dunnett’s multiple comparison test. To compare the two groups, the Mann–Whitney U test was applied for numerical data, and Fisher’s exact test was used for categorical data. Spearman’s rank correlation coefficient was used to verify the correlation between changes in the CAVI and inflammatory markers. Simple linear regression models were used to assess the relationship between changes in CAVI and patient characteristics, inflammatory markers, lipid variables, or HbA1c and screen for independent variables to be included in the multiple linear regression model. A backward stepwise multiple linear regression was conducted to build a candidate model. The final multiple regression model was presented and interpreted after performing model diagnostics. The level of significance was set at *p* < 0.05. 

## 3. Results

### 3.1. Patient Profile

Table 1 shows the demographic and clinical data of the patients. The mean age of the patients was 63.4 ± 18.2 years (mean ± standard deviation [SD]). The mean BMI was 21.7 ± 3.5. The mean CAVI of the patients was 8.8 ± 1.4. The mean initial daily dose of prednisolone was 37.3 ± 16.1 mg. Hypertension, diabetes mellitus, and dyslipidemia were observed in about one-fourth of the patients.

### 3.2. Changes in CAVI during Glucocorticoid Therapy

**Figure 2:**
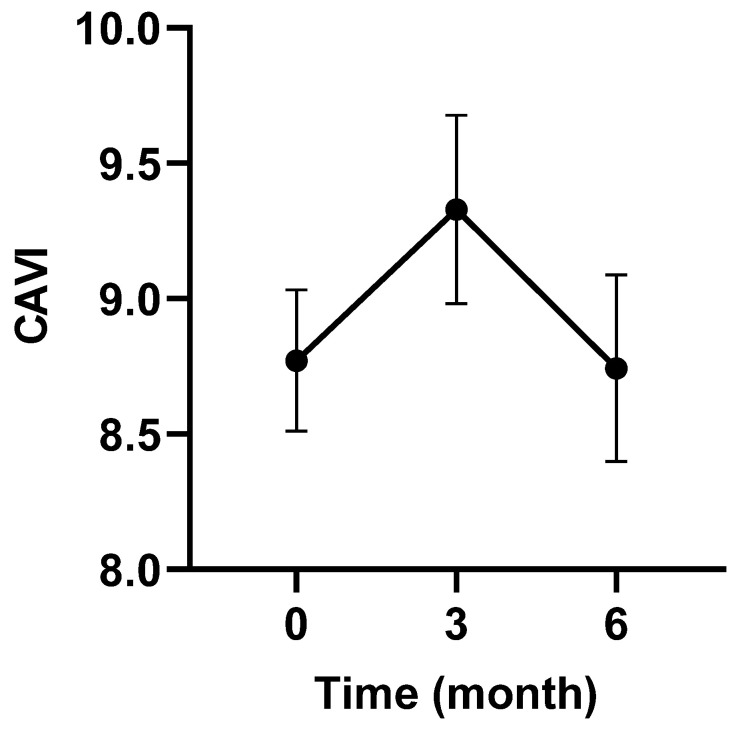
Changes in CAVI in all patients during glucocorticoid therapy. Data are presented as the mean ± standard deviation. Friedman’s test followed by Dunnett’s multiple comparison test were performed. CAVI, cardio-ankle vascular index; GC, glucocorticoid.

Figure 2 shows that GC therapy increased mean CAVI after 3 months, but it decreased to pretreatment levels after 6 months (mean ± SD; 8.8 ± 1.4, 9.3 ± 1.8, 8.7 ± 1.8). No significant difference was observed between the 3 months and 6 months compared to the pretreatment values.

### 3.3. Comparing the CAVI Changes between Patients over and Patients under 65 Years

Table 2 presents the demographic and clinical data of patients aged ≥65 years and those <65 years at baseline. Mean CAVI and serum CRP at baseline were significantly higher in patients >65 years than in those <65 years. The comorbidity of diabetes was significantly higher in patients over 65 years compared to patients under 65 years. No significant difference was observed in the mean initial GC dose between the two groups.

**Figure 3:**
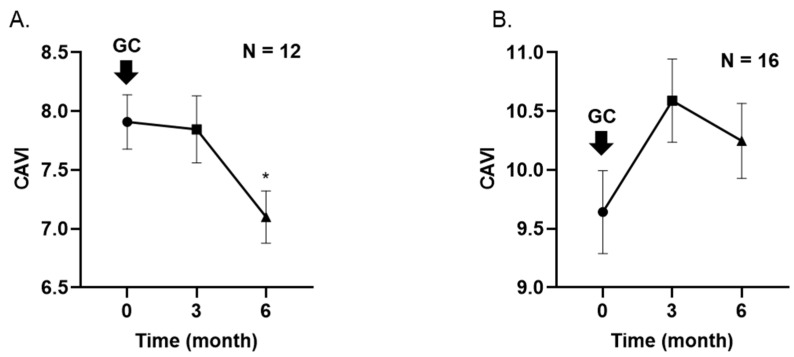
Changes in CAVI in patients under 65 years (**A**) and over 65 years (**B**) during glucocorticoid therapy. Data are expressed as the mean ± standard deviation. * indicates statistical significance (*p* < 0.05) by Friedman’s test followed by Dunnett’s multiple comparison test. CAVI, cardio-ankle vascular index; GC, glucocorticoid.

Figure 3A,B show that mean CAVI was significantly decreased in patients aged <65 years compared to baseline and after 6 months (7.9 ± 0.8 to 7.1 ± 0.8, *p* < 0.05), while mean CAVI increased in patients aged ≥65 years (9.6 ± 1.5 to 10.2 ± 1.4, *p* = 0.15).

### 3.4. Association between CAVI Change and Inflammation Markers at Baseline

**Figure 4:**
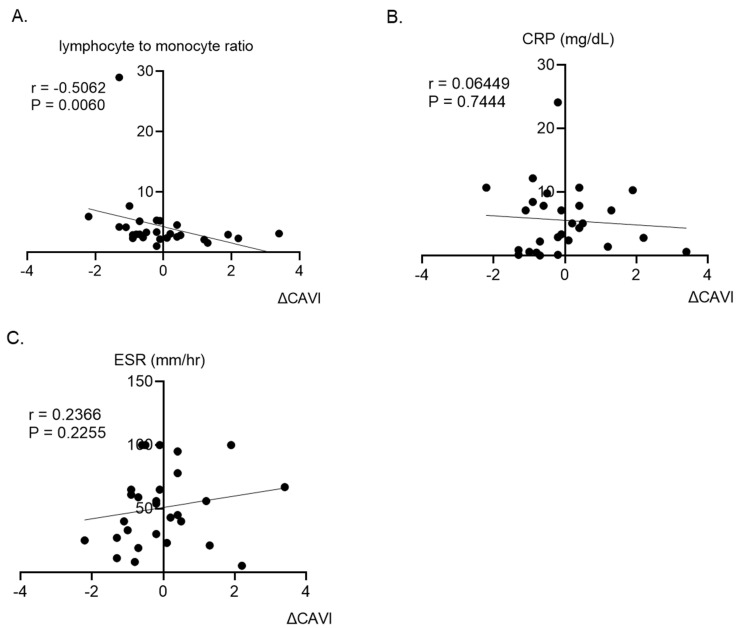
Correlation between changes in CAVI and lymphocyte-to-monocyte ratio (**A**). Correlation between changes in CAVI and CRP (**B**). Correlation between changes in CAVI and ESR (**C**). Relationships were assessed by Spearman’s rank correlation test. CAVI, cardio-ankle vascular index; CRP, C-reactive protein; ESR, erythrocyte sedimentation rate.

There was a significant negative correlation between the variation in CAVI values during 6 months of GC treatment and LMR at baseline (R = −0.5062, *p* = 0.006) (Figure 4A). In contrast, no correlation was observed between the variation in CAVI values and conventional inflammatory markers such as CRP (R = 0.06449, *p* = 0.7444) and erythrocyte sedimentation rate (R = 0.2366, *p* = 0.2255) (Figure 4A,B).

### 3.5. Multivariate Analysis of Factors Associated with CAVI Progression

We examined the independent influence of the CAVI changes in the patients with rheumatic diseases using multiple regression analyses adjusted for patient characteristics (age, sex, and BMI), prednisolone dose at baseline, inflammatory markers (LMR, CRP, and ESR), lipid variables (HDL-C, LDL-C, triglycerides), and HbA1c (Table 3). Factors significantly related to increased changes in CAVI based on the univariate analysis were old age and low LMR at baseline. The multivariate model also highlighted old age, high prednisolone dosage, and low baseline LMR as significantly related factors (Table 3).

## 4. Discussion

This is the first study to demonstrate changes in arterial stiffness assessed using CAVI during GC therapy in patients with rheumatic diseases. We demonstrated that CAVI increased after 3 months of GC therapy and then decreased to pretreatment levels after 6 months in patients with rheumatic diseases. CAVI increased after 3 months in patients >65 years and tended to decrease after 6 months of GC therapy, whereas CAVI decreased significantly in younger patients. Our results also imply that GC may normalize pressure wave reflections in patients with rheumatic diseases. Changes in CAVI levels were negatively correlated with LMR at baseline, and LMR was an independent factor associated with the progression of arterial stiffness. These results suggest that LMR may be an important predictor of the progression of arterial stiffness associated with rheumatic disease during GC treatment.

CAVI is a recently developed index of arterial stiffness calculated from the heart-ankle pulse wave velocity (PWV) adjusted for blood pressure by β, a stiffness parameter. Therefore, CAVI represents the stiffness of the arterial tree from the origin of the aorta to the ankle and is not influenced by blood pressure changes during measurement [13]. As body weight or blood pressure may change during GC treatment in patients with rheumatic diseases, arterial stiffness parameters independent of blood pressure may be clinically useful for monitoring changes in arterial stiffness during the follow-up period. CAVI may be a useful clinical tool for assessing changes in arterial stiffness in patients with rheumatic diseases undergoing GC treatment, whose blood pressure changes easily. In addition to being a reproducible marker of early atherosclerosis, CAVI has been reported to predict future CVD events [18]. In this study, the mean CAVI of our patients before GC treatment (8.8) was comparable to the cutoff CAVI (8.81) associated with coronary arteriosclerosis [19]. This result indicates that patients with various rheumatic diseases before GC treatment are already at high risk of CVD before GC treatment.

Sixty-four patients with low disease activity SLE (mean age 45 years, disease duration 15 years) had significantly increased CAVI compared with healthy controls (SLE vs. healthy controls: 7.3 vs. 6.3) [20]. Sato et al. [21] reported markedly higher CAVI in premenopausal patients with SLE than in participants with premenopausal control (7.5 vs. 6.4). CAVI tended to be higher in postmenopausal patients with SLE than in postmenopausal control participants (8.6 vs. 7.9). However, these studies differ from ours in that they report the results of CAVI in patients with SLE already undergoing GC treatment. Furthermore, these studies did not explore changes in CAVI before and after GC or immunosuppressive therapy. Kume et al. [22] investigated changes in CAVI before and after treatment with biological disease-modifying antirheumatic drugs in 64 patients with previously untreated active RA. CAVI in the patients significantly decreased from 10.7 to 9.8 before and after treatment with biological disease-modifying antirheumatic drugs. These findings support our results of arterial stiffening improvement with anti-inflammatory interventions, such as GC treatment.

We previously investigated the effects of nitroglycerin on arterial stiffness in patients with and without CAD using CAVI [23,24]. After nitroglycerin administration, the stiffness of the arteries measured by CAVI decreased in both healthy controls and patients with CAD, indicating that the response of arterial smooth muscle cells was preserved even in patients with CAD under medication. However, the time to maximum depression of CAVI in healthy controls was faster than that in patients with CAD. This indicates that the response of vascular smooth muscle to nitroglycerin administration was faster in healthy controls than in patients with CAD. The drugs administered differed. However, these findings support our results that CAVI decreased from baseline after GC treatment in younger patients compared with older patients.

LMR was first defined as a biomarker for infectious diseases and reflects the balance between the effector and the host [25]. Lymphocytes and monocytes are crucial components of innate and acquired immunity. Recently, LMR has gained recognition as a biomarker of inflammation associated with rheumatic diseases. Du et al. [26] published a study reporting the effectiveness of LMR as an inflammatory marker effective in disease activity evaluation in patients with RA and RA differentiated from other arthritic conditions. Wang et al. [27] revealed a decreased LMR in axial spondylarthrosis, an inflammatory arthritis affecting the spine, compared with healthy controls, especially in patients with axial spondylarthrosis and high X-ray stages. A study of LMR in patients with SLE demonstrated lower LMR in cases than in healthy controls, which indicated that LMR was significantly differentially expressed in patients with SLE and healthy controls [28]. This study revealed that LMR is a useful biomarker for predicting renal function and prognosis in patients with SLE. These studies have demonstrated the relationship between various aspects of rheumatic diseases and LMR as a novel systemic inflammatory marker.

Inflammation plays an important role in the initiation and progression of atherosclerotic processes [29]. Lymphocytes are involved in the regulatory pathways of the immune system [30], and inflammation increases lymphocyte apoptosis [31]. Kurtul et al. [32] demonstrated an association between LMR and the no-reflow phenomenon in patients who underwent primary percutaneous coronary intervention for ST-elevation myocardial infarction. Additionally, a relationship between LMR and bare-metal in-stent restenosis in patients with spontaneous coronary artery dissection has been reported [33]. This study suggests that LMR is an inflammatory biomarker contributing to atherosclerosis from onset to progression. Therefore, LMR has been proven to be an important inflammatory marker and a potential predictor of CVD risk [12]. However, the relationship between the LMR and CVD risk in patients with systemic rheumatic disease undergoing GC treatment has not yet been elucidated. We revealed a significant negative correlation between changes in CAVI during 6 months of GC treatment and baseline LMR in patients with rheumatic disease. Conventional inflammatory markers such as CRP and ESR have been extensively explored and are correlated with disease activity in rheumatic diseases [34]. No correlation was observed between changes in CAVI values and conventional inflammatory markers such as CRP and ESR. Hence, LMR was determined as a better inflammatory marker than conventional markers in terms of changes in CAVI values during GC treatment. In addition, multivariate analysis demonstrated that LMR was an independent factor associated with arterial stiffness progression. LMR may be a potentially available, easily calculable, and cost-effective parameter for arterial stiffness prognosis before GC therapy in rheumatic diseases.

### Study Limitations

This study has some limitations. As the study was performed at a single hospital with a retrospective observational design, patient selection bias may have affected the results. The study population was relatively small, and a larger number of patients is needed. Our study included patients with various rheumatic diseases. Although we analyzed the differences in CAVI between inflammatory diseases (e.g., RA) and non-inflammatory diseases (e.g., SLE), no difference was found in the variation in CAVI values between the two groups. Further research on individual diseases will be needed in the future. Additionally, we evaluated the effect of GC therapy for > 6 months on arterial stiffness in patients with rheumatic disease. However, the long-term influence of GC therapy on the progression of arterial stiffness in patients with rheumatic diseases remains unclear. Further multicenter, large-scale prospective studies are needed to strengthen our conclusions.

## 5. Conclusions

GC temporarily exacerbates arterial stiffness and then improves it in rheumatic diseases. Particularly in young patients, GC may improve arterial stiffness by reducing inflammation. CAVI is a useful clinical marker for evaluating arterial stiffness in patients with rheumatic diseases undergoing GC therapy. LMR may be a potentially available, easily calculable, and cost-effective parameter for arterial stiffness prognosis before GC therapy in rheumatic diseases. In conclusion, our findings suggest that patients with rheumatic diseases, especially those aged <65 years, may reduce vascular damage by receiving appropriate GC therapy to relieve arterial constriction.

## Figures and Tables

**Table 1:** Demographics and clinical data at the baseline of the study population.

	*N* = 28
Age (years)	63.4 ± 18.2
Female/male	16/12
Body mass index (kg/m^2^)	21.7 ± 3.5
Cardio-ankle vascular index	8.8 ± 1.4
Comorbidities	
Hypertension (%)	7 (25%)
Diabetes mellitus (%)	6 (21.4%)
Dyslipidemia (%)	9 (32.1%)
Current smoking (%)	4 (14.3%)
Ever smoked (%)	11 (39.3%)
Laboratory Data	
C-reactive protein (mg/dL)	4.7 ± 5.2
Erythrocyte sedimentation rate (mm/h)	50.9 ± 29.1
White blood cell (/μL)	8963 ± 3776
Lymphocytes (/μL)	1439 ± 394
Neutrophil (/μL)	6658 ± 3590
Monocytes (/μL)	463 ± 209
Lymphocytes/monocytes ratio	4.3 ± 5.0
Total cholesterol (mg/dL)	183.9 ± 44.1
High-density lipoprotein cholesterol (mg/dL)	110.0 ± 32.3
Low-density lipoprotein cholesterol (mg/dL)	47.6 ± 15.0
Triglycerides (mg/dL)	124.6 ± 88.6
HbA1c (%)	6.2 ± 0.7
Medications	
Initial prednisolone dose (mg/day)	37.3 ± 16.1
Antihypertensive agents (%)	7 (25.0%) (CCB 3, ARB 2, CCB+ARB 1)
Antidiabetic agents (%)	6 (21.4%)
Statins (%)	9 (32.1%)
Systemic Autoimmune Diseases	
Vasculitis syndrome	5 (17.9)
Polymyalgia rheumatica	5 (17.9)
Systemic lupus erythematosus	3 (10.7)
IgG4-related disease	3 (10.7)
Dermatomyositis/polymyositis	3 (10.7)
Adult-onset Still’s disease	3 (10.7)
RS3PE syndrome	2 (7.1)
Systemic Sclerosis	2 (7.1)
Rheumatoid arthritis	1 (3.6)
Relapsing polychondritis	1 (3.6)

Data are displayed as mean ± standard deviation and number (%), as appropriate. Abbreviations: ARB: angiotensin II receptor blocker, CCB: calcium channel blocker, RS3PE syndrome: remitting seronegative symmetrical synovitis with pitting edema.

**Table 2:** Demographics and clinical data for patients aged 65 years or older and patients younger than 65 years at baseline.

	Patients < 65 Years *N* = 12	Patients ≥ 65 Years *N* = 16	*p*-Value
Age (years)	45.1 ± 11.3	77.1 ± 6.4	<0.0001
Female/male	8/4	8/8	0.4589
Body mass index (kg/m^2^)	23.2 ± 2.9	21.4 ± 2.3	0.1555
Cardio-ankle vascular index	7.9 ± 0.8	9.4 ± 1.3	0.001
Comorbidities			
Hypertension (%)	1 (8.3%)	6 (37.5%)	0.3778
Diabetes mellitus (%)	0 (0%)	6 (37.5%)	0.0237
Dyslipidemia (%)	4 (33.3%)	5 (31.3%)	>0.9999
Current smoking (%)	1 (8.3%)	3 (18.8%)	0.5983
Ever smoked (%)	7 (58.3%)	4 (25.0%)	0.1212
Laboratory Data			
C-reactive protein (mg/dL)	2.4 ± 3.5	7.1 ± 5.4	0.0017
Erythrocyte sedimentation rate (mm/h)	33.7 ± 16.5	63.9 ± 29.0	
White blood cell (/μL)	8964 ± 3888	9578 ± 3784	0.347
Lymphocytes (/μL)	1439 ± 377	1940 ± 964	0.8281
Neutrophil (/μL)	6658 ± 3701	6628 ± 3440	0.8639
Monocytes (/μL)	463 ± 227	542 ± 285	0.7058
Lymphocytes/monocytes ratio	3.2 ± 1.7	5.2 ± 6.4	0.1183
Total cholesterol (mg/dL)	196.6 ± 34.1	174.4 ± 48.2	0.0973
High-density lipoprotein cholesterol (mg/dL)	46.6 ± 17.6	48.3 ± 13.0	0.1243
Low-density lipoprotein cholesterol (mg/dL)	99.7 ± 40.6	117.2 ± 22.6	0.6927
Triglycerides (mg/dL)	125.0 ± 117.1	124.4 ± 59.0	0.9859
HbA1c (%)	6.1 ± 0.3	6.5 ± 0.9	0.0598
Medications			
Initial prednisolone dose (mg/day)	40.4 ± 16.1	35.6 ± 15.8	0.3678
Antihypertensive agents (%)	1 (8.3%) (CCB 1)	6 (37.5%) (CCB 2, ARB 2, CCB+ARB 1)	0.3778
Antidiabetic agents (%)	0 (0%)	6	0.0237
Statins (%)	4 (33.3%)	5	>0.9999
Systemic Autoimmune Diseases			
Vasculitis syndrome	3 (17.9)	2 (17.9)	0.6239
Polymyalgia rheumatica	0 (0)	5 (0)	0.0525
Systemic lupus erythematosus	3 (10.7)	3 (10.7)	>0.9999
IgG4-related disease	0 (10.7)	3 (10.7)	0.2381
Dermatomyositis/polymyositis	0 (10.7)	3 (10.7)	0.2381
Adult-onset Still disease	3 (10.7)	0 (10.7)	0.0672
RS3PE syndrome	0 (7.1)	2 (7.1)	0.4921
Systemic sclerosis	1 (7.1)	1 (7.1)	>0.9999
Rheumatoid arthritis	1 (3.6)	0 (3.6)	0.4286
Relapsing polychondritis	1 (3.6)	0 (3.6)	0.4286

Data are presented as mean ± standard deviation and number (%), as appropriate. Abbreviations: ARB: angiotensin II receptor blocker, CCB: calcium channel blocker, RS3PE syndrome: remitting seronegative symmetrical synovitis with pitting edema.

**Table 3:** Univariate and multivariate analysis of characteristics associated with changes in CAVI.

Characteristics	ΔCAVI			
	Univariate Model		Multivariate Model	
	β	*p*-Value	β	*p*-Value
Age	0.0343	0.0036	0.0345	0.0021
Female	0.2979	0.5228	−0.6192	0.0646
Body mass index	−0.0321	0.6943	−0.0460	0.4130
Prednisolone dose (mg/day) at baseline	0.0030	0.8359	0.0223	0.0455
lymphocyte-to-monocyte ratio	−0.3267	0.0363	−0.3741	0.0030
C-reactive protein (mg/dL)	−0.0183	0.6815	−0.0629	0.091
Erythrocyte sedimentation rate (mm/h)	0.0072	0.3592	0.0065	0.2932
High-density lipoprotein cholesterol (mg/dL)	0.0038	0.7623	0.0074	0.5083
Low-density lipoprotein cholesterol (mg/dL)	−0.0079	0.2488	0.011	0.1595
Triglycerides (mg/dL)	0.0003	0.8937	−0.0016	0.4056
HbA1c (%)	0.0410	0.8961	0.2197	0.2940

ΔCAVI: the number of changes in CAVI. Abbreviations: CAVI: cardio-ankle vascular index.

## Data Availability

The original contributions presented in the study are included in the article, and further inquiries can be directed to the corresponding author.
